# Long-term surveillance of sulfate-reducing bacteria in highly saline industrial wastewater evaporation ponds

**DOI:** 10.1186/1746-1448-5-2

**Published:** 2009-02-18

**Authors:** Eitan Ben-Dov, Ariel Kushmaro, Asher Brenner

**Affiliations:** 1 Department of Biotechnology Engineering, Ben-Gurion University of the Negev, PO Box 653, Be'er-Sheva, 84105, Israel; 2 Achva Academic College, MP Shikmim, 79800, Israel; 3 National Institute for Biotechnology of the Negev, Ben-Gurion University of the Negev, PO Box 653, Be'er-Sheva 84105, Israel; 4 Unit of Environmental Engineering, Ben-Gurion University of the Negev, PO Box 653, Be'er-Sheva, 84105, Israel

## Abstract

Abundance and seasonal dynamics of sulfate-reducing bacteria (SRB), in general, and of extreme halophilic SRB (belonging to *Desulfocella halophila*) in particular, were examined in highly saline industrial wastewater evaporation ponds over a forty one month period. Industrial wastewater was sampled and the presence of SRB was determined by quantitative real-time PCR (qPCR) with a set of primers designed to amplify the dissimilatory sulfite reductase (*dsrA*) gene. SRB displayed higher abundance during the summer (10^6^–10^8 ^targets ml^-1^) and lower abundance from the autumn-spring (10^3^–10^5 ^targets ml^-1^). However, addition of concentrated dissolved organic matter into the evaporation ponds during winter immediately resulted in a proliferation of SRB, despite the lower wastewater temperature (12–14°C). These results indicate that the qPCR approach can be used for rapid measurement of SRB to provide valuable information about the abundance of SRB in harsh environments, such as highly saline industrial wastewaters. Low level of H_2_S has been maintained over five years, which indicates a possible inhibition of SRB activity, following artificial salination (≈16% w/v of NaCl) of wastewater evaporation ponds, despite SRB reproduction being detected by qPCR.

## Findings

Sulfate-reducing bacteria (SRB) are anaerobic microorganisms that use sulfate as an electron acceptor. They are known to grow both heterotrophically, relying on small organic molecules, and autotrophically, using H_2 _as the electron donor and CO_2 _as the carbon source [1]. However, several studies have demonstrated that certain species of SRB are not only able to tolerate high concentrations of oxygen but can also utilize oxygen as a terminal electron acceptor [2]. SRBs are known to be present in the complex consortia of microorganisms involved in the anaerobic digestion processes used in municipal and industrial wastewater treatment. Furthermore, sulfate reduction may account for up to 50% of the mineralization of organic matter in aerobic wastewater treatment systems [3]. A major drawback of sulfate reduction in wastewater treatment is the production of the toxic odorant H_2_S, which in addition, is an agent that significantly enhances microbially-mediated corrosion of treatment facilities [1]. This is especially true in the oil industry, where sulfate reduction causes severe problems, including souring of oil and gas deposits [4].

Dissimilatory sulfate reduction occurs up to quite high salt concentrations. Black sediments are often found on the bottom of salt lakes and saltern ponds approaching NaCl saturation [5,6]. Some culturable halophilic sulfate reducers, such as *Desulfovibrio halophilus*, *Desulfocella halophila*, *Desulfovibrio oxyclinae *and *Desulfohalobium retbaense*, may grow from 18 up to 24% NaCl concentration at the upper limit [7-10]. The *dsrAB *genes which encode dissimilatory sulfite reductase, the key enzyme in dissimilatory sulfate reduction, can be used as a phylogenetic marker for identification of SRB [11]. These genes are found in all known sulfate-reducing prokaryotes [12]. Thus, *dsrAB *from all sulfate-reducing lineages can be targeted by a single set of conserved primers or using specific primers from variable regions of *dsrAB*.

In this study, a pair of universal PCR primers for the functional gene *dsrA *[13] and two specific sets of primers for *dsrA *of halophilic SRB, were designed and used for long-term surveillance of SRB populations within five highly saline industrial wastewater evaporation ponds by quantitative real-time PCR (qPCR). These ponds are the final treatment stage of a combined wastewater stream contributed to by several chemical plants (manufacturing various pesticides, pharmaceuticals, aliphatic and aromatic halogens) at the Ramat-Hovav industrial park in the Negev desert, Israel [14]. Organic matter concentration in the wastewater stream is 2–2.5 gC/l (on the basis of total organic carbon measure), of which over 30% reaches the evaporation ponds. Receiving a mixture of saline, high strength industrial wastewater, these ponds offer a unique habitat for various microorganisms [15]. In order to reduce the foul odors emitted by the ponds and, in particular, the H_2_S that results from SRB activity, salinity of the evaporation ponds was artificially raised (August–October 2003) from an initial 3–7% to a final concentration of about 12% (w/v) by addition of NaCl [16].

Total genomic DNA of wastewater samples was extracted [13] from obtained pellets (derived from 30 ml samples) using the MoBio Power Soil DNA isolation kit (MoBio Laboratories, Solana Beach, CA). The pair of PCR primers (DSR1F and RH3-dsr-R) that specifically detect and quantify SRB was used as previously described [13]. The measured values were transformed to targets per milliliter of wastewater.

**Figure 1:**
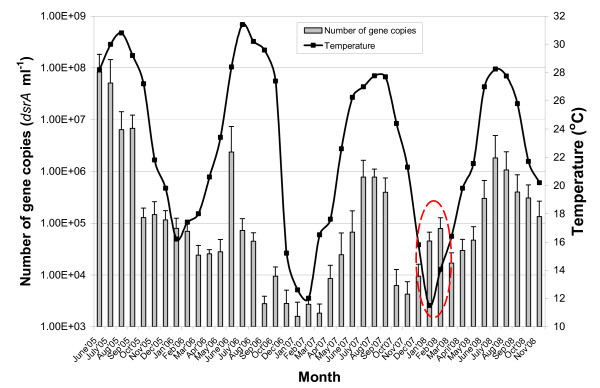
**Seasonal evolution of SRBs in highly saline industrial wastewater evaporation ponds**. Samples at 6 meter depth were collected monthly from five industrial wastewater evaporation ponds, over a forty one month period. The abundance of SRB was quantified by qPCR, with the temperature at same depths also being recorded. Bar heights represent means based on average samples of all five industrial wastewater ponds, while error bars represent standard deviation.

The levels and seasonal dynamics of SRB present in the complex environment of highly saline and concentrated evaporation ponds demonstrated temperature-dependent behavior, with higher abundance being detected during summer (about 10^6^–10^8 ^targets ml^-1^, temperature range from 28 to 31°C) and lower levels being noted during the winter months of November-March (about 10^3^–10^5 ^targets ml^-1^, temperature range from 12 to 18°C) (Figure. 1). Recently, similar high *dsr *gene copy numbers (as measured by qPCR) of up to 10^8 ^SRB cells ml^-1 ^were detected in hypersaline soda lakes of the Kulunda Steppe in southeastern Siberia in Russia, where total salt concentration ranges from 50 to 500 g liter^-1 ^[6]. Over next three summers (2006–2008), the abundance of SRB in the evaporation ponds decreased to levels of 10^6 ^targets ml^-1^, with the average temperature being about 28°C. The relatively low levels of SRB detected during the summers, relative to that level measured in the summer of 2005 (10^8 ^targets ml^-1^), might be attributed to subsequent increases in salinity (due to natural evaporation) up to about 14–18% (w/v), to diminished flow distribution between the ponds, and to the reduction of organic load following the gradual application of treatment processes by the various industrial plants. On the other hand, during January-February of 2008, an unusual increase in SRB levels was observed (up to 10^5 ^targets ml^-1^), despite lower temperature that ranged from 11.5 to 14°C (Figure. 1; marked by a dashed red ellipse). Further investigation revealed that at the end of December, 2007, a massive load of concentrated organic matter and biomass residuals from a bioreactor (that had been cleaned) was poured into the evaporation ponds, immediately contributing to proliferation of SRB. In March, 2008, the concentration of SRB decreased again (to 1.7 × 10^4 ^targets ml^-1^), within to the usual range seen in non-summer months. Seasonal dynamics of SRB follow temperature changes, as well as the single occurrence of an increase in SRB levels following an organic load increase (Figure. 1), indicate that our qPCR approach is applicable for SRB monitoring in harsh environmental niches, such as highly saline industrial wastewaters. Similar seasonal dynamics of SRB in mudflats of the Seine estuary, i.e. with higher abundance and activity being detected during the early summer, was demonstrated by Leloup *et al*. [17], using competitive PCR analysis of the *dsrAB *genes. These authors also suggested that SRB appeared to be mainly controlled by physical-chemical parameters (e.g. temperature and dissolved organic carbon concentration) and the topographic evolution of the mudflat (i.e. erosion/deposit erosion).

Tree highly specific PCR primers were developed for quantifying extreme halophilic SRB levels. The one forward (RH-halo-1F) and two reverse (RH-halo-1R and RH-halo-2R) primers (Table 1) for *dsrA *were designed based on multiple alignments of *dsrA *of *Desulfocella halophila *DSM 11763 (AF418200), *D. halophila *(AF388211) and *dsrA *sequences (e.g. EF052891, EF052876, EF052909 [13]) obtained from the SRB community present in the industrial wastewater evaporation ponds. The two primer pairs, RH-halo-1F/RH-halo-1R and RH-halo-1F/RH-halo-2R, generated specific DNA products of 144 bp and 187 bp found within the *dsrA *sequence, respectively (Table 1). All primer sequences were verified by running an actual, as well as virtual, PCR, with the amplifications being analyzed for expected product sizes, matching (as well as mis-matching) positions within the *dsrA*, and primer dimer formation, using a Amplify version 1.0, developed by William Engles, Department of Genetics, University of Wisconsin. All primer sequences were examined by the BLASTN (for a short input sequence), to confirm the absence of any significant homology to other known DNA sequences. Specific PCR products, obtained using genomic DNA extracted from industrial wastewater samples as template provide reproducible distinct melting points of 78°C and 80°C for the RH-halo-1F/RH-halo-1R pair (amplifying a 144 bp fragment) and the RH-halo-1F/RH-halo-2R pair (amplifying a 187 bp product) primers respectively. Negative controls and amplification of DNA from a non-SRB strain (i.e. *E*.*coli*) did not yield any PCR products using either primer set.

**Table 1:** Oligonucleotides used for real-time PCR to amplify halophilic *dsrA *genes closely related to *Desulfocella halophila*.

Primer pair^a^	Sequence (5'-3')^b^	Primer binding site^c^	Product size (bp)
RH-halo-1F	GTTCTTcTtGGTACAAGAACAGA	192–214	144
RH-halo-1R	GCATGAGTATTCACATCTT	317–335	
RH-halo-1F	GTTCTTcTtGGTACAAGAACAGA	192–214	187
RH-halo-2R	GGAATTCCTGTGTCAAgAAaTGA	356–378	

**a**. (F) and (R) correspond to forvard and reverse primers, respectively.

**b**. Bases that do not match appropriate sequences are shown as lowercase letters.

**c**. Positions within the *Desulfocella halophila *DSM 11763 (AF418200; Friedrich, 2002) *dsrA *open reading frame.

**Figure 2:**
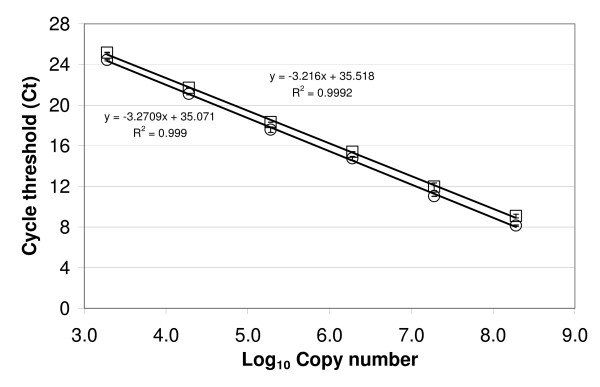
**Standard curves for SRB *dsrA *gene levels attained using two sets of primers, namely RH-halo-1F and RH-halo-1R (open circles), and RH-halo-1F and RH-halo-2R (open squares)**. Threshold cycle values (Ct) are plotted against 1.9 × 10^3 ^to 1.9 × 10^8 ^*dsrA *copies per assay, contained in a 10-fold dilution of the plasmid harboring the *dsrA *sequence. Data points represent the average of duplicate measurements, while error bars indicate standard deviation.

Using plasmids harboring *dsrA *gene sequences from local SRB community (RH.dsrA-208-49-18 (DQ662504) or RH.dsrA-206-1 (EF052876)), we generated standard curves by qPCR, using duplicate serial dilutions of known amounts of circular plasmid DNA. Linearity and reproducibility of the standard curves were tested using the RH-halo-1F/RH-halo-1R and RH-halo-1F/RH-halo-2R primer pairs. The standard curve for the *dsrA *gene with the two sets of primers, behaved in a linear manner between 1.9 × 10^3 ^and 1.9 × 10^8 ^copies per assay (0.95 × 10^2 ^to 9.5 × 10^6 ^copies ml^-1^) with slopes of -3.27 (R^2 ^value > 0.99) and -3.22 (R^2 ^value > 0.99), respectively (Figure. 2). Plasmid DNA was also mixed with filtrated (0.1 mm) and purified (as mentioned above) industrial wastewater and compared with plasmid DNA from a pure culture of *E. coli*. No significant differences in slope were observed (data not shown), ensuring the validity our approach to overcome inhibition.

**Figure 3:**
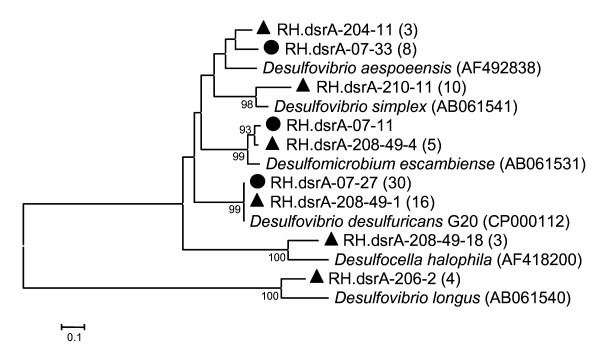
**Phylogenetic comparison of *dsrA *gene sequences retrieved from industrial wastewater evaporation ponds in 2005 and 2007**. Geometric forms indicate the sequences according to the sampling date: black triangle, 2005 and black circle, 2007. Tree was constructed by the Neighbor-Joining method [19] with the Mega package [21] using partial *dsrA *sequences. The numbers in parentheses indicate the total number of similar clones on the basis of ≥ 97% identity for each representative sequence. The bar represents ten substitutions per 100 nucleotide positions. Bootstrap probabilities [20] are indicated at branch nodes.

Examination of the amount of extreme halophilic SRB within the highly saline industrial wastewater evaporation ponds, quantified by qPCR with two sets of specific primers (RH-halo-1F/RH-halo-1R and RH-halo-1F/RH-halo-2R), revealed low levels, from tens to few hundreds copies of halophilic *dsrA *ml^-1^. The low level of halophilic SRB (belonging to *D. halophila*), observed over five years after artificial salination (August–October 2003), may result from a suppression of halophilic SRB reproduction due to increasing salinity (14–18%) and decreasing biogenic organic matter loads. Known culturable halophilic SRB are display optimal growth at NaCl concentrations, ranging from 4 to 10% [7-10]. However, the steady state conditions detected could be interrupted in future by halophilic SRB species with higher optimal salinity growth requirements. The upper limit of salt concentration for halophilic and halotolerant sulfate reducers appear to be 24% NaCl, where these SRB are usually incompletely degrade organic compounds due to bioenergetic considerations [5]. Nevertheless, the more general pair of specific primers (i.e. DSR1F and RH3-dsr-R) enables quantification of seasonal SRB fluctuations in durable highly saline industrial wastewater evaporation ponds. These primers were derived based on aligned consensus regions of *dsrA *and display specificity to a wide range of SRB genera, such as *Desulfovibrio*, *Desulfomonas*, *Desulfatibacillus*, *Desulfomicrobium*, *Desulfobacterium*, *Desulfosarcina*, *Desulfonema*, *Desulfofaba*, *Desulfomusa*, *Desulfotignum*, *Desulfotomaculum*, *Desulfacinum*, *Desulfonatronum*, *Desulfoarculus*, *Desulfovirga *and others [13]. Due to artificial salination, significant reduction in H_2_S concentrations (from ppm to ppb levels) in the ponds area was observed [16]. This low level of H_2_S has been maintained over five years, which indicates a possible inhibition of SRB activity in the highly saline (14–18% NaCl) wastewater evaporation ponds, despite SRB reproduction, as inferred from increasing of *dsrA *copy numbers, as detected by qPCR. In addition, phylogenetic comparison (the sequences compared were cloned and sequenced as previously described [13,16]) of 41 *dsrA *sequences (EF052874–EF052921) (amplified using DSR1F/DSR4R primers) and 39 *dsrA *sequences (FJ231216–FJ231254) (amplified using DSR1F/DSR10R primers [18]) respectively retrieved from the industrial wastewater evaporation ponds in 2005 and 2007 (both after salination) did not indicate any significant exchange in the SRB population (Figure. 3; phylogenetic tree was constructed by Neighbor-Joining method [19], bootstrap probabilities [20] with the Mega package [21]). Nevertheless, the diversity of SRB from 2007 was lower than in 2005. Indeed, sequences with relative homology to *D. halophila*, *Desulfovibrio longus *and *Desulfovibrio simplex *were not found, although qPCR performed with specific primers continuously detected basal levels of *D. halophila*. Our qPCR approach indicates the presence of a dynamic SRB community in these extreme ecosystems, with seasonal fluctuations related to temperature changes and to pronounced waste disposal incidents. This method has proven itself to be a reliable means of monitoring SRB in a straight manner so as to provide early warning for possible sulfide production and subsequent prevention of potential corrosion or odor nuisances.

## Abbreviations

SRB: Sulfate-reducing bacteria; qPCR: quantitative real-time PCR; *dsrA*: dissimilatory sulfIte reductase; w/v: weight per volume.

## Competing interests

The authors declare that they have no competing interests.

## Authors' contributions

EB-D participated in the design of the experiments, carried out isolation of total genomic DNA, sequencing of *dsrA* genes and phylogenetic analysis, performed the statistical analysis and drafted the manuscript. AK participated in the conception and design of study, interpretation of data. AB participated in the interpretation of data. All the authors drafted, read and approved the final manuscript.
